# Seasonal, Aspect and Elevational Effects on Auchenorrhyncha Communities in Taibai Mountain, China

**DOI:** 10.3390/insects17060586

**Published:** 2026-06-04

**Authors:** Luwei Liu, Yusen Du, Xiuyun He, Haoyue Zhang, Chunni Zhang, Wu Dai

**Affiliations:** State Key Laboratory for Crop Stress Resistance and High-Efficiency Production, Key Laboratory of Plant Protection Resources and Pest Management of Ministry of Education, College of Plant Protection, Northwest A&F University, Yangling 712100, China; liuluwei@nwafu.edu.cn (L.L.); yusendu@nwafu.edu.cn (Y.D.); hexiuyun@nwafu.edu.cn (X.H.); zhanghaoyue@nwafu.edu.cn (H.Z.)

**Keywords:** auchenorrhyncha, taibai mountain, species diversity, systematic composition, vertical distribution pattern

## Abstract

Forest biomes exhibit considerable spatial heterogeneity in insect diversity, hosting complex and dynamic insect assemblages. Auchenorrhyncha, a highly diverse infraorder of herbivorous hemipterans, demonstrates strong associations with native flora and exhibits predictable responses to environmental changes, rendering this group an ideal bioindicator for ecosystem health assessments. While the utility of these insects has been recognized in grassland ecosystems, their utility for monitoring montane forests remains underexplored. This study investigates the spatiotemporal and elevational distribution patterns of Auchenorrhyncha on Taibai Mountain, a critical biodiversity hotspot within China’s Qinling Range. Systematic field surveys conducted along a continuous elevational gradient (1100 to 3700 m a.s.l.) documented a total of 124 species belonging to 80 genera and 12 families. Notably, species richness was significantly higher on the southern slope (95 species/60 genera) than on the northern slope (71 species/51 genera). Clear elevational stratification was observed: Cicadellidae and Aphrophoridae were widely distributed, whereas Fulgoroidea and Membracidae were restricted elevations below the *Betula utilis* zones, and Cicadidae occurred exclusively in *Quercus variabilis* and *Q. aliena* forests. Community diversity peaked at mid-elevations, consistent with the mid-domain effect, and was highest in *Q. aliena* forests. Community similarity was greater within the same forest type across different slopes than between adjacent elevational zones on the same slope. Seasonal analysis revealed peak diversity in July, with the southern slope consistently supporting higher diversity across all sampling periods. These findings not only establish essential baseline data but also confirm the potential of Auchenorrhyncha as effective bioindicators for monitoring biodiversity and ecological changes in complex montane ecosystems.

## Simple Summary

This study focuses on the use of Auchenorrhyncha—a group of highly diverse and environmentally sensitive sap-feeding insects including leafhoppers, planthoppers, spittlebugs and cicadas—as ecological indicators for assessing biodiversity patterns in mountain forests. We conducted a comprehensive survey on Taibai Mountain in the Qinling Range, a key biodiversity region in central China, to examine how their diversity varies across elevation gradients, between contrasting northern and southern slopes, and throughout the growing season. In total, field sampling recorded 124 species from 12 families, with significantly greater richness recorded on the southern slope. Community composition showed strong elevational stratification, with certain insect families restricted to specific forest zones, while overall diversity peaked at mid-elevations—a pattern consistent with ecological theory. Furthermore, forest type strongly shaped community structure, with oak forests supporting particularly high insect diversity. Seasonal dynamics indicated that species richness peaked in July. These findings provide novel insights into the spatial distribution and seasonal dynamics of Auchenorrhyncha in montane forests and confirm their practical value as reliable bioindicators for long-term ecological monitoring and conservation planning in complex mountain landscapes.

## 1. Introduction

Biodiversity encompasses the variety of plants, animals and microorganisms that inhabit the ecosystems of the planet, with each organism performing unique ecological functions that collectively sustain the integrity and resilience of biological systems [1]. As the essential foundation for human well-being, climate regulation, and ecological stability, biodiversity conservation is an urgent imperative for sustainable development and planetary health, making it a global priority [2,3,4].

Climate change represents one of the most significant threats to global biodiversity in the 21st century, with profound implications for ecosystem structure and function. Rising global temperatures, altered precipitation patterns, and increased frequency of extreme weather events are driving shifts in species distributions, phenology, and interactions worldwide [5]. In terrestrial ecosystems, insects represent a taxonomically dominant and ecologically pivotal group, comprising an estimated 75–80% of all known animal species [6,7]. This extraordinary diversity is matched by their exceptional ecological plasticity and functional variety [7]. Consequently, their high sensitivity to environmental change makes them invaluable bioindicators for monitoring ecosystem health [8,9].

The functional contributions of insects are irreplaceable. Phytophagous insects regulate plant metabolism and influence pathogen dynamics [10,11]; pollinators, responsible for the reproduction of nearly 90% of flowering plants, are critical for crop yield and floral diversity [12]; while predators provide essential biological control services by regulating prey populations [13]. Alarmingly, a growing body of evidence points to precipitous global insect declines. Meta-analyses suggest local insect abundances may be falling by up to 2.5% annually, a rate exceeding that observed in many vertebrate groups, signaling a potential crisis in ecosystem functioning [14,15]. Climate change acts as a potent accelerant of these declines, compounding existing pressures by forcing range shifts, creating phenological mismatches (e.g., between insect pollinators and plant flowering), and destabilizing community networks [16,17,18,19]. In response, establishing robust, long-term monitoring programs with standardized protocols has become crucial for tracking insect diversity trends, deciphering their environmental drivers, and generating reliable indicators for evidence-based conservation [12,20,21,22,23].

Insect assemblages, particularly in structurally complex forests ecosystems, often exhibit pronounced spatial heterogeneity and form dynamic communities that respond sensitively to environmental gradients [24]. Within these assemblages, Auchenorrhyncha, a suborder of Hemiptera, have been recognized as valuable bioindicators of habitat quality. Their indicator value is particularly evident in grassland-forest ecotones, where strong host-plant dependence and high habitat specificity make them especially sensitive to subtle environmental variation [25]. Auchenorrhyncha (Hemiptera), comprising the infraorders Cicadomorpha and Fulgoromorpha, presents an exemplary model for such biodiversity research. It is a well-classified group characterized by high species richness, broad distribution, and varied ecological roles, from sap-feeding to vectoring plant pathogens. Historically, research on Auchenorrhyncha diversity has illuminated how geographic isolation and landscape history contribute to phylogenetic divergence and dispersal patterns [26,27,28]. Studies have mapped diversity across taxa and habitats, identifying centers of endemism linked to ancient biogeographical processes [29,30]. Despite this foundational work, a significant research bias persists. Available literature remains disproportionately focused on the agricultural impacts of a few pest species and their management [31], leaving a substantial gap in understanding the full scope of the diversity, spatial distribution patterns, and nuanced ecological roles of these insects. Many prior studies also lack rigorous statistical frameworks to quantify the relationship between species richness and key environmental variables, and analyses focusing on patterns of endemism are particularly scarce.

The Qinling Mountains, forming a formidable barrier marking the boundary between the Oriental and Palearctic realms in central China, are recognized as a critical biodiversity hotspot. Straddling distinct climatic and biogeographic zones, they act as a vital ecological corridor and a climatic divide between northern and southern China. This region offers a unique natural laboratory due to its dramatic altitudinal gradients, pronounced habitat heterogeneity, and steep climatic transitions [32]. Taibai Mountain, the highest peak of the range, hosts one of the most complete vertical vegetation spectra in the region, fostering exceptional habitat diversity and a rich insect fauna [32]. Previous entomological surveys on Taibai Mountain have focused on various groups, including as Diptera, Orthoptera, Formicidae and Lepidoptera, consistently highlighting how the contrasting environmental gradients between the northern and southern slopes shape community composition [33,34,35,36,37,38,39]. The distribution of biodiversity along such gradients is often discussed in the context of Stevens’ extension of Rapoport’s rule to altitude, which predicts that species’ elevational ranges increase with altitude due to greater environmental tolerances in high-elevation taxa. This theory has stimulated numerous studies on how invertebrate species richness and range sizes shift across mountain systems globally [40,41,42]. For instance, studies on butterflies have demonstrated distinct assemblage structure on each slope, correlated with differences in temperature and humidity [38]. However, comprehensive diversity studies at finer taxonomic resolutions, such as the suborder level, remain limited. There is a clear need for improved survey design, broader taxonomic coverage, standardized data methodologies, and more sophisticated analytical frameworks to accurately assess the status and conservation requirements of insect biodiversity in this hotspot [43].

The present study constructs a comprehensive database of Auchenorrhyncha species for Taibai Mountain by integrating extensive field investigations with a thorough review of historical literature. We focus explicitly on comparing the northern and southern slopes, synthesizing data from multiple sources including the Insect Museum of Northwest A&F University, the National Specimen Information Infrastructure, and relevant publications. Through systematic biogeographic analyses, we aim to assess the distribution patterns, diversity characteristics, and endemicity of Auchenorrhyncha across this major mountain gradient. Our objective is to generate a foundational dataset that advances the understanding of hemipteran diversity in the Qinling Mountains and provides a scientific basis for regional and national conservation and ecological research.

## 2. Materials and Methods

### 2.1. Study Region

**Figure 1:**
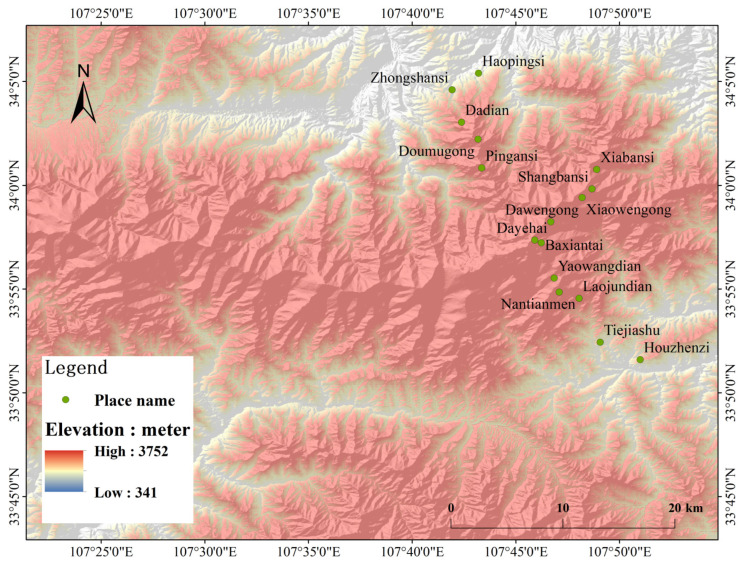
Distribution of sample points on the survey line across Taibai Mountain.

The study area is situated within the central Qinling Mountains of Shaanxi Province. As illustrated in Figure 1, the surveyed area is geographically defined by Laojun Mountain (Zhouzhi County) to the east, Aoshan Mountain (Taibai County) to the west, Longdonggou (Zhouzhi County) to the south, and Heihuguan (Yingtou Town, Mei County) to the north, spanning coordinates 107°22′25″–107°51′30″ E and 33°49′30″–34°05′35″ N. This region encompasses Taibai Mountain, the highest peak of the Qinling range and the most prominent mountain in eastern mainland China. This prominent elevation gradient creates a compressed spectrum of climatic zones and vegetation types, from warm-temperate deciduous broadleaf forests at lower elevations to alpine scrub and meadows near the summit. The pronounced environmental contrast between the northern (drier, sunnier) and southern (more humid, shaded) slopes establishes an ideal natural setting for investigating how topography and climate shape insect distribution and community assembly.

### 2.2. Data Collection

To comprehensively assess Auchenorrhyncha diversity along the complex topographic and climatic gradients of Taibai Mountain, a stratified sampling strategy was adopted based on elevation and slope aspect. Field surveys were conducted during the peak activity period of Auchenorrhyncha, from May to September 2022. Each month, both the southern and northern slopes were surveyed for 20 days. On the southern slope, a primary survey route was established from Houzhenzi to Baxiantai, that covered a continuous elevational gradient from 1100 to 3700 m above sea level. This gradient was divided into 14 contiguous 200-m elevational bands, and the midpoint of each band was used as the representative altitude in the regression analyses. Along this route, 7 sampling transects were established to cover major habitat transitions across the elevational gradient. On the northern slope, surveys covered a comparable elevational range from 1000 to 3700 m above sea level, which was divided into 15 continuous 200 m elevational bands. Sampling was conducted along two independent routes, one from Haopingsi and the other from Xiabansi, with a total of 10 transects converging at Baxiantai (see Table 1 for main site information). This dual-route design was used to account for potential microhabitat heterogeneity on the northern and southern slopes and to improve the representative of sampling. Across all transects and elevational bands, Auchenorrhyncha specimens were collected using a standardized sweep-net protocol. To ensure comparability of abundance data among sampling sites, the number of sweeps was kept consistent within each sampling unit and applied under comparable vegetation conditions, thereby minimizing collector bias.

### 2.3. Analysis Methods

A multi-faceted analytical framework was used to quantify and compare Auchenorrhyncha community structure along elevational and slope gradients.

Alpha Diversity: To characterize community diversity within each sampled site, four complementary indices were calculated: Shannon–Wiener index (H’), Pielou’s evenness index (J), Simpson’s dominance index (C), and Margalef’s richness index (R). These indices were selected because they capture different aspects of community structure, including overall diversity, relative evenness, dominance concentration, and species richness corrected for sample size.

Beta Diversity: To evaluate compositional similarity among communities from different slopes and elevational bands, beta diversity was assessed using the Jaccard similarity coefficient (Cj), which is based on species presence-absence data. This index was chosen because it provides a straightforward measure of species turnover and shared taxa among sampling units, independent of abundance variation.

Comparative statistical analyses. Differences in abundance, species richness, and diversity indices among slopes and elevational bands were tested using one-way analysis of variance (ANOVA) when the assumptions of normality and homogeneity of variance were satisfied. One-way ANOVA was selected because it is appropriate for comparing the means of multiple independent groups. When significant differences were detected, Tukey’s honestly significant difference (HSD) test was conducted for post hoc multiple comparisons to identify which groups differed significantly while controlling for Type I error.

When the assumptions of normality or homogeneity of variance were violated, non-parametric alternatives were applied. Specifically, the Kruskal–Wallis test was used for comparisons among multiple groups, followed by Dunn’s post hoc test for pairwise comparisons where appropriate.

For comparisons involving only two groups, such as between the northern and southern slopes at the same elevational level, independent-samples *t*-tests were used when parametric assumptions were met. When these assumptions were not satisfied, the Mann–Whitney U test was employed as a non-parametric alternative. These tests were selected because they are suitable for assessing differences between two independent groups.

Correlation and regression analyses. Pearson correlation analysis was used to examine relationships among abundance, species richness, and diversity indices, as well as their associations with elevation. Pearson’s correlation was chosen because it is appropriate for testing linear relationships between continuous variables. In addition, regression analysis was conducted to evaluate diversity patterns along the elevational gradient. For these analyses, the midpoint of each elevational band was used as the representative altitude. Regression analysis was selected to identify the direction and strength of diversity responses to changes in elevation.

Multivariate analyses. To explore patterns of community similarity and spatial differentiation among sampling sites, cluster analysis and Principal Component Analysis (PCA) were performed based on species composition data. These methods were chosen because they are effective for revealing grouping patterns among communities and visualizing compositional differences in reduced-dimensional space.

All statistical computations were performed using SPSS 21.0 (IBM Corp, Armonk, NY, USA), while graphical visualizations of diversity patterns along environmental gradients were generated with Origin 2018 (OriginLab, Northampton, MA, USA). This integrated approach allowed us to move beyond simple species lists and rigorously analyze the structure and spatial patterning of Auchenorrhyncha assemblages in relation to the mountain’s physical gradients.

## 3. Results

### 3.1. Characteristics of Auchenorrhyncha in Different Forest Belts

A total of 124 species of Auchenorrhyncha, belonging to 80 genera and 12 families, were recorded in the survey. Among these, 95 species from 60 genera and 8 families were collected on the southern slope, while 71 species from 51 genera and 9 families were found on the northern slope. Notable distribution patterns were observed among families: Cicadellidae and Aphrophoridae occurred across all forest zones of Taibai Mountain, whereas Fulgoroidea and Membracidae are restricted to elevations below the *Betula utilis* zone. In contrast, Cicadidae were exclusively recorded in the *Quercus variabilis* and *Q. aliena* belts.

#### 3.1.1. Alpha Diversity Patterns of Auchenorrhyncha

**Figure 2:**
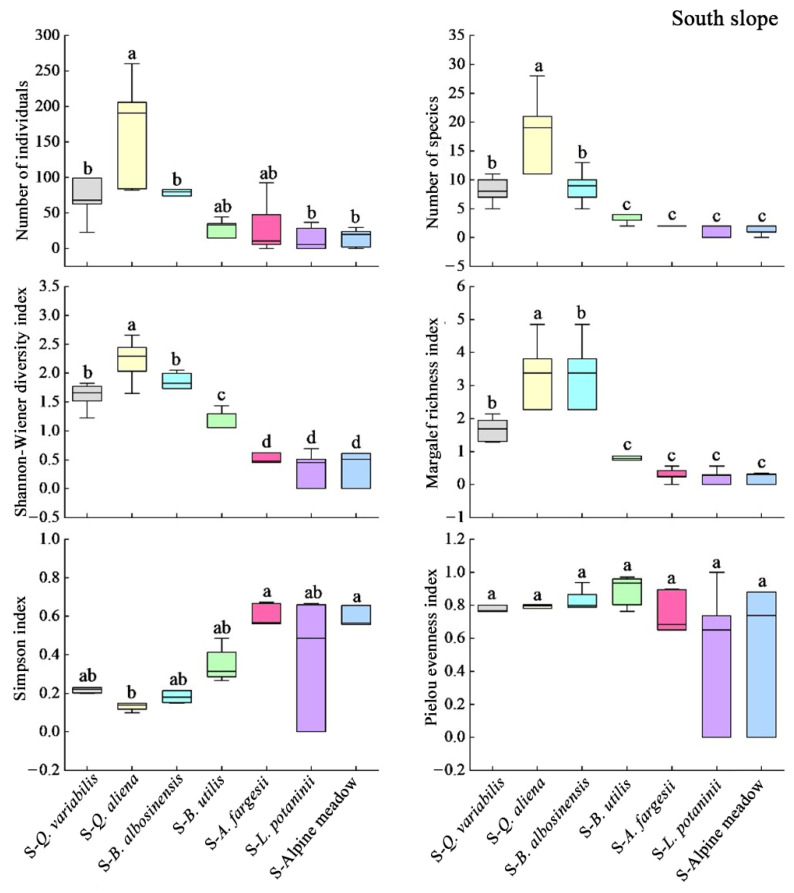
Species diversity of Auchenorrhyncha in different forest belts on the southern slope of Taibai Mountain. Forest belts on the x-axis are arranged in ascending order of elevation, and S—indicates the southern slopes. Note: The colored box represents the interquartile range (IQR). The central horizontal line is the median. The whiskers represent the most extreme data point within 1.5 × IQR above and below the box. Specifically, the upper whisker extends to the largest observed data point that falls within 1.5 times the IQR above the third quartile, while the lower whisker extends to the smallest observed data point within 1.5 times the IQR below the first quartile. Different letters (a, b, c, …) indicate significant differences among groups according to Tukey’s HSD test following ANOVA (*p* < 0.05).

As shown in Figure 2, on the southern slope of Taibai Mountain, the abundance of Auchenorrhyncha in *Q. aliena* forests was significantly higher than that in *Q. variabilis*, *B. utilis*, *Larix potaninii*, and Alpine meadow vegetation (*p* < 0.05). Regarding species diversity, the *Q. aliena* forests also exhibited significantly greater values for the Shannon–Wiener diversity index and Margalef richness index compared to other forest types. The *Q. variabilis* and *B. utilis* forests showed intermediate diversity, which was significantly higher than remaining four forest belts (including *L. potaninii* and Alpine meadow), where no significant differences were detected among the latter (*p* < 0.05). Notably, the *Q. aliena* forest had the lowest Simpson dominance index, indicating a more equitable species distribution and reduced dominance by a few taxa. In contrast, Pielou’s evenness index did not differ significantly among the forest belts, suggesting similar levels of species abundance uniformity across communities.

**Figure 3:**
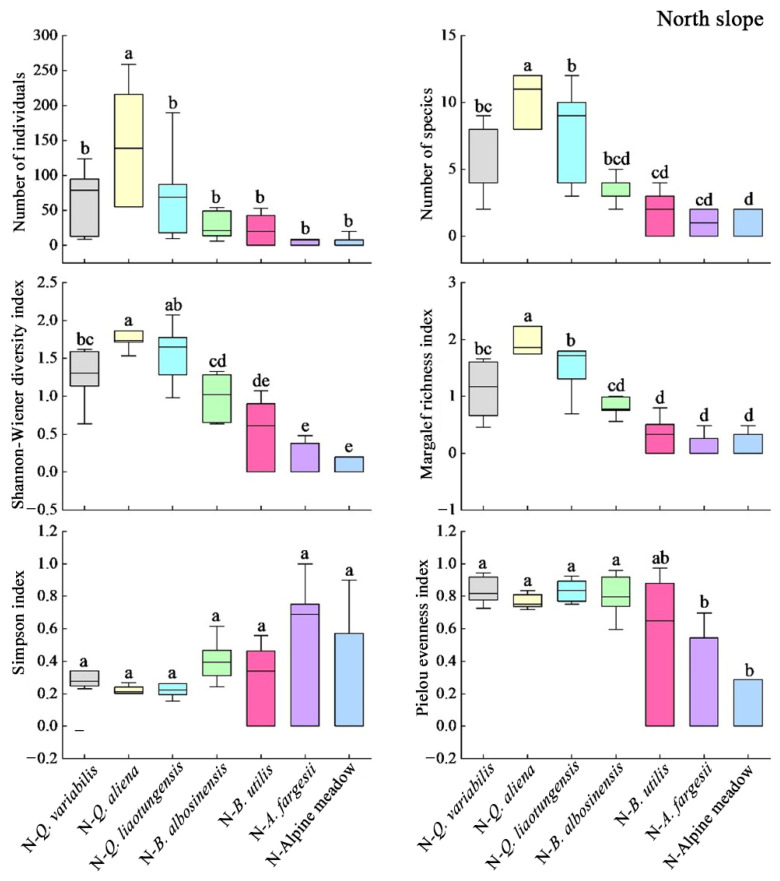
Species diversity of Auchenorrhyncha in different forest belts on the northern slope of Taibai Mountain. Forest belts on the x-axis are arranged in ascending order of elevation, and N-indicates the northern slopes. Different letters (a, b, c, …) indicate significant differences among groups according to Tukey’s HSD test following ANOVA (*p* < 0.05).

On the northern slope (Figure 3), Auchenorrhyncha abundance in *Q. aliena* forests also significantly exceeded that of the other six forest belts (*p* < 0.05). In terms of biodiversity metrics, *Q. aliena* forests exhibited significantly higher species richness, Shannon–Wiener index, and Margalef index values than *Q. variabilis* and *B. utilis* forests. The latter two forest types, in turn, surpassed the remaining four—including *Abies fargesii* and Alpine meadow—in these metrics (*p* < 0.05), with *A. fargesii* and Alpine meadow consistently showing the lowest values. No significant differences were observed in the Simpson dominance index across all northern slope forest types. Pielou’s evenness index did not vary significantly among *Q. variabilis*, *Q. aliena*, *Q. liaotungensis*, and *B. utilis* forests; however, these four forest types exhibited significantly higher evenness than *A. fargesii* and Alpine meadow (*p* < 0.05).

#### 3.1.2. Correlation and Vertical Distribution Patterns

Correlation analysis showed significant relationships among Auchenorrhyncha community indices on both slopes (Table 2 and Table 3). The number of individuals (IND), species richness (SPE), Shannon–Wiener diversity index (H′), and Margalef’s richness index (R) were all significantly and positively correlated with one another on the southern slope of Taibai Mountain (Table 2). In particular, species richness was strongly correlated with the number of individuals (*r* = 0.930, *p* < 0.01), Shannon–Wiener diversity (*r* = 0.900, *p* < 0.01), and Margalef’s richness index (*r* = 0.990, *p* < 0.01). Likewise, H′ was significantly positively correlated with R (*r* = 0.940, *p* < 0.01). In contrast, Simpson’s dominance index (C) showed significant negative correlations with SPE (*r* = −0.420, *p* < 0.05), H′ (*r* = −0.430, *p* < 0.01), and R (*r* = −0.450, *p* < 0.01). Pielou’s evenness index (J) was significantly positively correlated with H′ (*r* = 0.570, *p* < 0.01) and weakly correlated with R (*r* = 0.360, *p* < 0.05), but showed no significant correlation with IND, SPE, or C.

Correlation analysis of data from the northern slope of Taibai Mountain showed significant positive relationships among the number of individuals (IND), species richness (SPE), Shannon–Wiener diversity index (H′), and Margalef’s richness index (R) (Table 3). Species richness was strongly positively correlated with IND (*r* = 0.890, *p* < 0.01), H′ (*r* = 0.910, *p* < 0.01), and R (*r* = 0.980, *p* < 0.01). Similarly, H′ was significantly positively correlated with IND (*r* = 0.780, *p* < 0.01) and R (*r* = 0.940, *p* < 0.01). Simpson’s dominance index (C) showed weak negative correlations with IND, SPE, H′, and R, but none of these relationships were statistically significant. Pielou’s evenness index (J) was significantly positively correlated with IND (*r* = 0.380, *p* < 0.05), SPE (*r* = 0.490, *p* < 0.01), H′ (*r* = 0.740, *p* < 0.01), and R (*r* = 0.560, *p* < 0.01), whereas its correlation with C was positive but not significant.

**Figure 4:**
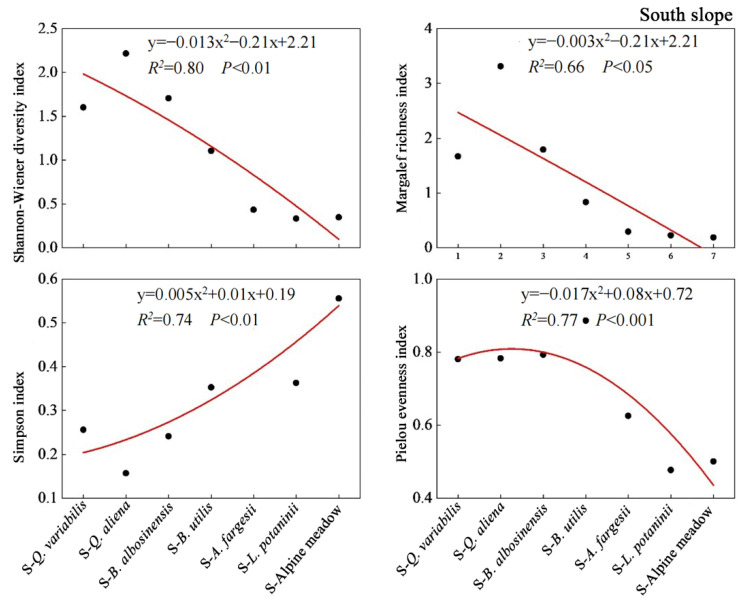
Curves fitting of the vertical distribution patterns of Shannon–Wiener, Margalef, Simpson, and Pielou diversity indices on the southern slope of Taibai Mountain. Forest belts on the x-axis are arranged in ascending order of elevation, and S- indicates the southern slopes.

**Figure 5:**
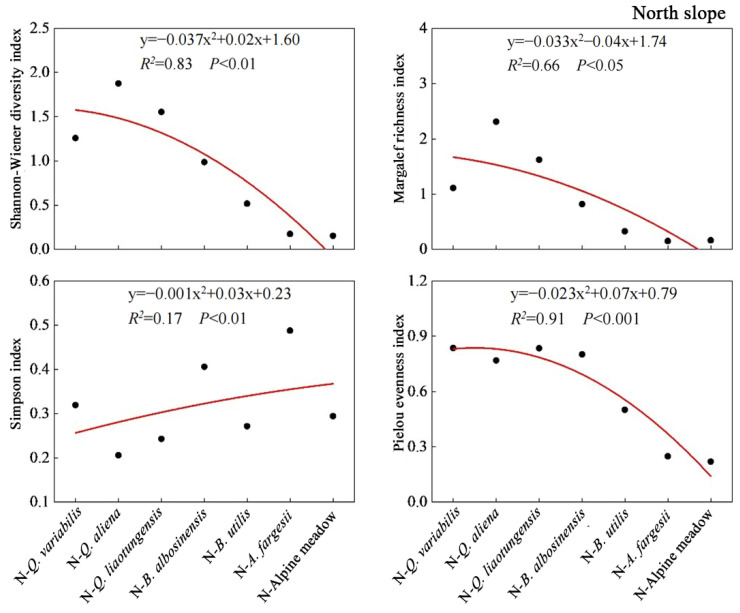
Curves fitting of the vertical distribution patterns of Shannon–Wiener, Margalef, Simpson, and Pielou diversity indices on the northern slope of Taibai Mountain. Forest belts on the x-axis are arranged in ascending order of elevation, and N- indicates the northern slopes.

Quadratic regression models effectively captured the vertical distribution patterns of diversity indices along the elevational gradient. On the southern slope (Figure 4), significant fits were obtained for the Shannon–Wiener index (R^2^ = 0.80, F_(2,4)_ = 8.14, *p* < 0.01), Margalef richness index (R^2^ = 0.66, F_(2,4)_ = 3.86, *p* < 0.05), Simpson index (R^2^ = 0.74, F_(2,4)_ =5.91, *p* < 0.01), and Pielou evenness index (R^2^ = 0.77, F_(2,4)_ = 6.88, *p* < 0.01). Similarly, on the northern slope (Figure 5), quadratic models provided strong fits for the Shannon–Wiener index (R^2^ = 0.83, F_(2,4)_ = 9.51, *p* < 0.01), Margalef richness index (R^2^ = 0.66, F_(2,4)_ = 3.94, *p* < 0.05), Simpson index (R^2^ = 0.17, F_(2,4)_ =0.40, *p* < 0.01), and Pielou evenness index (R^2^ = 0.91, F_(2,4)_ = 19.43, *p* < 0.01).

#### 3.1.3. Community Similarity of Auchenorrhyncha

As summarized in Table 4, the Jaccard similarity coefficients of Auchenorrhyncha communities among forest belts on the southern slope of Taibai Mountain exhibited clear ecological patterns. Similarity values ranged widely, with several pairs of forest types exhibiting moderate similarity (0.25–0.5), including Alpine meadow-*Larix potaninii*, Alpine meadow-*Abies fargesii*, *Larix potaninii*-*Abies fargesii*, *Abies fargesii*-*Betula utilis*, *Betula utilis*-*Betula utilis*, *Betula utilis*-*Quercus aliena*, and *Quercus aliena*-*Quercus variabilis*. Among these, the Alpine meadow and *A. fargesii* forest shared the highest similarity (0.44). In contrast, the Alpine meadow showed extremely low similarity with *Q. variabilis* and *Q. aliena* forests (coefficient = 0.02 in both cases), with only one species shared in each comparison. The remaining forest belt pairs showed low similarity (coefficients < 0.25). Notably, adjacent forest belts generally exhibited higher similarity than non-adjacent ones, reflecting relatively steady community turnover with change in elevation.

The Jaccard similarity coefficients for Auchenorrhyncha communities among different forest belts on the northern slope of Taibai Mountain are summarized in Table 5. The similarity was low (coefficents between 0.25–0.5) for three pairwise comparisons: Alpine meadow vs. *A. fargesii*, *Q. liaotungensis* vs. *Q. aliena*, and *Q. aliena* vs. *Q. variabilis*. Among these, the *Q. aliena* and *Q. variabilis* shared the highest number of species (23), corresponding to the maximum similarity coefficient of 0.45 within this low-similarity range. In contrast, the similarity coefficient for all other forest belt comparisons fell below 0.25, indicating extremely low community similarity. Notably, the Alpine meadow shared no species with either the *Q. liaotungensis* or the *Q. variabilis* forests, resulting in a similarity coefficient of zero for these pairs.

#### 3.1.4. Principal Component and Cluster Analysis

**Figure 6:**
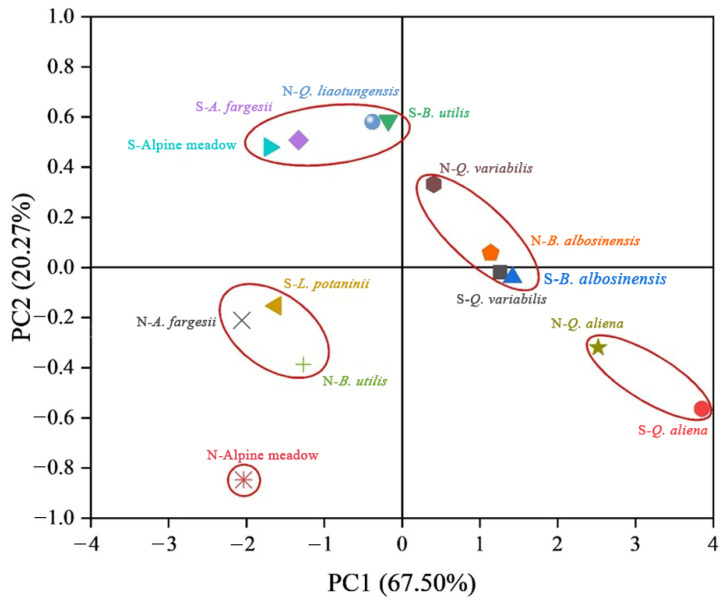
Principal component analysis (PCA) of Auchenorrhyncha community composition across different forest belts on the northern and southern slopes of Taibai Mountain. S- and N- indicate the southern and northern slopes, respectively.

Principal component analysis of Auchenorrhyncha species composition across seven forest types on the southern and northern slopes of Taibai Mountain clearly segregated the communities into five distinct groups (Figure 6). *Q. aliena* forests from both slopes (S-*Q. aliena* and N-*Q. aliena*), which supported the highest species richness, clustered together, representing mid-elevation habitats with high diversity. A second group comprised *Q. variabilis* and *B. utilis* forests from both slopes (S-*Q. variabilis*, N-*Q. variabilis*, S-*B. utilis* and N-*B. utilis*), reflecting similar elevations and species composition. A third group, representing mid- to high-elevation habitats, included N-*A. fargesii*, N-*B. utilis*, and S-*L. potaninii*. The fourth group, characterized by low species diversity and a predominance of high-elevation species, consisted of S-Alpine meadow, S-*A. fargesii*, N-*Q. liaotungensis* and S-*B. utilis*. The N-Alpine meadow formed a separate group, distinguished by its low overall Auchenorrhyncha abundance and minimal species richness.

**Figure 7:**
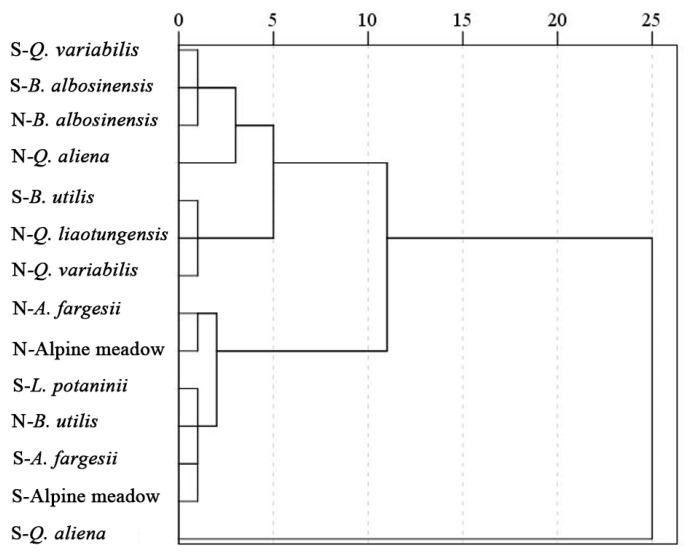
Cluster analysis of Auchenorrhyncha community composition across different forest belts on the northern and southern slopes of Taibai Mountain. S- and N- indicate the southern and northern slopes, respectively.

Cluster analysis based on squared Euclidean distance further classified the Auchenorrhyncha communities into three primary groups at a linkage distance of 5 (Figure 7). The high-diversity group included seven forest belts: S-*Q. variabilis*, S-*B. utilis*, N-*B. utilis*, N-*Q. aliena*, S-*B. utilis*, N-*Q. liaotungensis*, and N-*Q. variabilis*. The low-diversity group consisted of six belts: N-*A. fargesii*, N-Alpine meadow, S-Alpine meadow, N-*B. utilis*, S-*A. fargesii*, and N-Alpine meadow. The S-*Q. aliena* forest was grouped independently, consistent with its uniquely high values in abundance, species richness, Shannon–Wiener diversity, and Margalef richness indices, as well as its relatively uniform species abundance distribution.

### 3.2. Diversity Analysis Across Sampling Months

#### 3.2.1. Monthly Variations in Alpha Diversity

**Figure 8:**
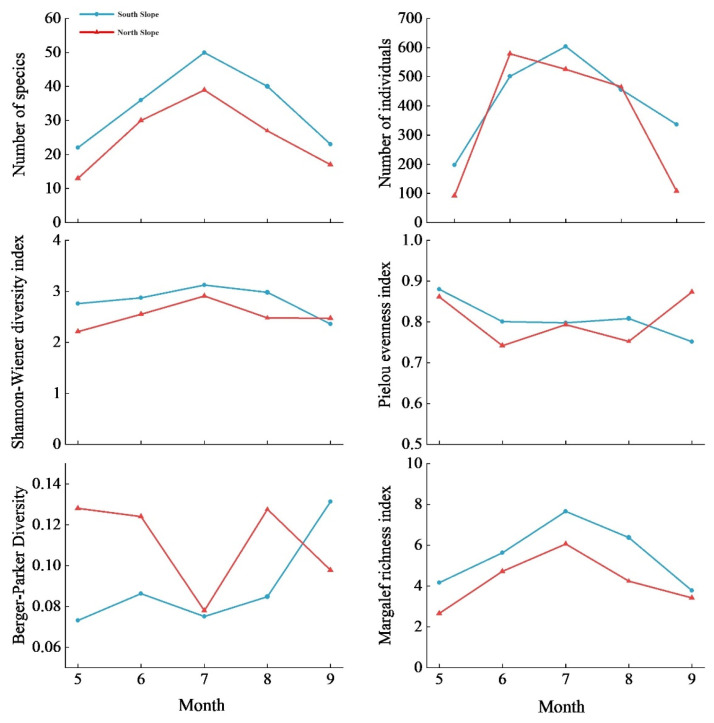
Temporal dynamics of species numbers, individual numbers, the diversity indices of Shannon–Wiener, Simpson, Margalef and Pielou of Auchenorrhyncha on the southern and northern slopes of Taibai Mountain.

As shown in Figure 8, the species richness, individual abundance, Shannon–Wiener diversity index and Margalef richness index of Auchenorrhyncha on both northern and southern slopes exhibited similar temporal trends, increasing steadily from May to a distinct peak in July, followed by a subsequent decline. Across all months, the southern slope consistently demonstrated higher values in these diversity metrics compared to the northern slope. The Pielou evenness index remained relatively stable on both slopes throughout the sampling period, indicating consistently uniform species abundance distributions within monthly assemblages. In contrast, the Simpson dominance index displayed slope-specific seasonal patterns: on the southern slope, dominance decreased from May to July then increased through September (September > June > August > July > May), while the northern slope showed highest dominance in May, decreasing through July before increasing again (May > August > June > September > July).

#### 3.2.2. Temporal Turnover in Community Composition

Jaccard similarity coefficients revealed distinct temporal patterns in community turnover on both slopes (Table 6 and Table 7). On the southern slope, low similarity (0.25–0.50) was observed between May–September, June–August, July–August, and August–September. The July–August pair exhibited the highest similarity (0.32) with 21 shared species, indicating minimal community turnover during this late-summer period. Very low similarity (0–0.25) characterized May–June, May–July, May–August, June–July, June–September, and July–September pairs, with the May–June combination showing the lowest similarity (0.09, 5 shared species). Overall, May communities displayed the greatest distinctness from other months, potentially due to lower overall richness, while August communities showed stronger similarity to those of adjacent months, particularly September.

Northern slope communities showed parallel but distinct patterns (Table 7). Low similarity (0.25–0.50) was observed among June–July, June–August, and July–August pairs, with June–August showing the highest similarity (0.33, 14 shared species). Very low similarity (0–0.25) characterized all other monthly combinations, with August–September being most dissimilar (0.13, 5 shared species). The summer months (June, July, August) formed a coherent temporal group with mutually high similarities, while May and September communities showed pronounced distinctness from other sampling periods.

## 4. Discussion

Based on species richness, abundance, Shannon–Wiener diversity index, and Margalef richness index analyses, Auchenorrhyncha communities exhibited maximum diversity in mid-altitude regions of Taibai Mountain, demonstrating a characteristic mid-peak pattern along the elevational gradient. This altitudinal distribution pattern has been widely documented across various mountain ecosystems and biological taxa [44,45,46]. The observed mid-domain effect, where species richness peaks in central elevation ranges due to geometric constraints of mountain topography, provides a primary explanation for this phenomenon. Comparable mid-peak distribution patterns have been reported for other insect groups across global mountain systems: butterfly species richness in Tanzania’s Uluguru Mountain (tropical Africa) peaked at 2050 m elevation [47], while pollinator-plant communities in San Francisco showed maximum richness in mixed conifer forests at intermediate elevations [48]. Consistent with these findings, exotic beetle assemblages on the eastern slope of Balang Mountain (eastern Tibetan Plateau) displayed peak diversity between 2400–2600 m [49]. These parallel observations across disparate geographic regions and taxonomic groups suggest that the Auchenorrhyncha communities of Taibai Mountain exhibit patterns of altitudinal zonation similar to those of other insect groups in various mountain regions of the world. Similar hump-shaped patterns have been observed in diverse taxa, including ants in the Pyrenees and South Africa [40,50], geometrid moths in the Andes and Costa Rica [41,51], and ground spiders in Crete [42], all showing a diversity peak at intermediate elevations where environmental conditions are optimized. The congruence between our findings and established biogeographic patterns implies that mid-elevation optimization of environmental conditions (e.g., temperature-humidity balance, vegetation complexity, and reduced edge effects) may collectively drive this characteristic mid-peak distribution in insect communities.

Taibai Mountain features a parallel north–south arrangement of gullies and valleys. Under the influence of the regional north–south airflow, the vertical vegetation zonation is broadly similar between slopes, which contributes to analogous altitudinal diversity patterns in many insect groups [52]. Cluster analysis of Auchenorrhyncha communities along the elevation gradient revealed that species diversity was significantly higher in broad-leaved forests than in coniferous forests. This pattern corresponds with the vertical distribution of forest types, suggesting a synergistic relationship and possible co-evolution between Auchenorrhyncha assemblages and their host plant communities [53].

Broad-leaved forest zones—such as those dominated by *Quercus variabilis* and *Quercus aliena* in the low-elevation areas—are characterized by a mosaic of artificial and secondary forests with heterogeneous stand structure and high floristic complexity. These zones also represent some of the most favorable habitats in the Qinling Mountains in terms of temperature, moisture, and soil conditions, which collectively support the highest recorded diversity of Auchenorrhyncha, with Typhlocybinae being the dominant subfamily.

The *Betula utilis* forest, situated in the mid-elevation rainy belt, exhibits high ecological adaptability. It forms an ecotonal zone connecting lower broadleaved forests and upper fir forests, eventually developing into mixed stands with complex vegetation structure. This transitional forest type supports higher Auchenorrhyncha species diversity than adjacent uniform forest types.

In contrast, high-altitude habitats above 2600 m, such as Abies forests and coniferous scrub, are characterized by simplified vegetation—primarily shrubby growth—coupled with low temperatures and strong winds [54]. These conditions limit insect diversity and abundance, resulting in relatively depauperate Auchenorrhyncha communities dominated by Cicadellinae and Evacanthinae.

Notably, stand richness was negatively correlated with precipitation and positively correlated with temperature. The variation in Auchenorrhyncha species richness along moisture and thermal gradients closely mirrored that of forest stand richness [55], underscoring the tight coupling between insect assemblages and microclimatic–vegetation structure. As noted in previous studies of herbivore communities [56], above-ground insects like Auchenorrhyncha are more directly exposed to surface climate and vegetation shifts along elevation than below-ground communities, which explains the high sensitivity of Auchenorrhyncha to thermal gradients and forest stand richness.

Climate change and human disturbance, as major divers of global biodiversity loss, are increasingly disrupting seasonal patterns and habitat structures worldwide [57,58]. Rising temperatures associated with climate change are expected to shift species distributions along elevation gradients, whereby cold-adapted species may be gradually replaced by those adapted to warm conditions [5,59,60]. Compared with the species survey conducted by Cao et al. in high-altitude regions from 2013 to 2015 [37], our findings suggest that some Auchenorrhyncha species are already experiencing population declines or local replacement. This trend underscores the importance of prioritizing biodiversity conservation in high-altitude ecosystems.

While many high-elevation insect species are often considered specialists with narrow ecological niches [61,62], Stevens extension of Rapoport’s elevational rule, suggesting that species at higher altitudes may actually possess broader environmental tolerances to survive more climatic variability. This increased tolerance could lead to wider elevational ranges for high-altitude taxa. However, our observations of relatively depauperate and specialized Auchenorrhyncha communities above 2600 m suggest that extreme physiological constraints—such as low temperatures and simplified vegetation—may still limit the range of such assemblages, as seen in other mountain systems where the Rapoport effect is not universally supported [63,64]. Specifically, recent studies on Orthoptera [65] and madicolous insects [66] have also failed to find a typical Rapoport effect, suggesting that high-altitude specialists may be restricted by specific habitat boundaries and host-plant availability rather than possessing broader tolerances. This contradicts the ‘rescue hypothesis’ associated with Rapoport’s rule and emphasizes the vulnerability of high-elevation Auchenorrhyncha to environmental filtering [63,64]. These taxa, often characterized by limited dispersal ability, have persisted through major climatic fluctuations since at least the last glacial cycle [67], with mountains serving as critical refugia during periods of range expansion and contraction [68].

In North America and Europe, high-altitude species adapted to cold environments are increasingly vulnerable to habitat reduction, physiological stress, and phenological mismatches, leading to population declines or local extinctions [5,69]. For instance, butterflies and bees that overwinter as eggs and rely on specific high-altitude host plants have experienced significant declines in recent decades due to climate warming [70,71]. Concurrently, we anticipate that many low- and mid-elevation species, which exhibit a preference for warmer conditions, will shift their distributions upward [63]. Such elevational shifts are likely to parallel those observed in plant communities under climate change [72,73].

Our analysis of the temporal variation in Auchenorrhyncha communities—based on species number, individual abundance, Shannon–Wiener diversity index, and Margalef richness index—revealed a consistent peak in diversity during July across both slopes of Taibai Mountain. Moreover, a comparative analysis between slopes demonstrated that the southern slope sustained significantly higher values across all these metrics than the northern slope. We posit that this observed pattern is primarily driven by the more favorable thermal and hydric conditions (e.g., higher temperatures and precipitation) on the southern slope [74].

## 5. Conclusions

The Auchenorrhyncha community in Taibai Mountain exhibited a distinct mid-elevation peak in species richness along the elevational gradient, consistent with the mid-domain effect. Species diversity peaked in the mid- to low-elevation *Quercus aliena* zone, followed by the *Betula utilis* and *Quercus variabilis* zones. The lowest diversity was observed in high-altitude fir forests and Alpine meadow.

Overall, the southern slope supported higher Auchenorrhyncha diversity than the northern slope. Community similarity was higher within the same forest zone between slopes than among different forest zones on the same slopes, indicating that vegetation type is a stronger determinant of species composition than slope aspect.

On both slopes, Auchenorrhyncha diversity peaked in July, followed by June and August. In each corresponding month, species diversity was consistently higher on the southern slope than on the northern slope, reflecting the combined effects of microclimate and habitat heterogeneity.

## Figures and Tables

**Table 1:** Geographic locations and vegetation distribution of the main survey sites.

Habitat	Sample Area	Forest Zone	Longitude	Latitude	Altitude/m
Deciduous broad-leaved forest	Haopingsi	*Quercus variabilis*	107°43′12.16″	34°05′03.22″	1200
	Houzhenzi	*Quercus variabilis*	107°51′13.21″	33°51′36.33″	1350
	Zhongshansi	*Quercus aliena*	107°41′55.78″	34°04′36.34″	1440
	Shangbansi	*Quercus aliena*	107°48′40.40″	33°59′49.77″	1800
	Dadian	*Quercus liaotungensis*	107°42′22.80″	34°03′02.24″	2300
	Tiejiashu	*Quercus aliena*	107°49′03.93″	33°52′26.17″	1575
Deciduous leaflet forest	Sanhegong	*Quercus aliena*	107°48′09.11″	33°53′24.08″	2000
	Laojundain	*Betula utilis*	107°48′02.93″	33°54′33.02″	2710
	Doumugong	*Betula albosinensis*	107°43′10.93″	34°02′13.54″	2600
Coniferous forest	Pingansi	*Abies fargesii*	107°43′21.57″	33°00′50.21″	2750
	Xiabansi	*Abies fargesii*	107°48′53.82″	34°00′45.85″	2800
	Nantianmen	*Larix potaninii*	107°47′05.72″	33°54′51.01″	3100
	Yaowangdian	*Larix potaninii*	107°46′50.99″	33°55′31.79″	3130
Alpine meadow	Dawengong	Alpine meadow	107°46′41.52″	33°58′14.10″	3550
	Baxiantai	Alpine meadow	107°46′13.80″	33°57′13.62″	3767
	Dayehai	Alpine meadow	107°45′54.97″	33°57′21.47″	3600
	Xiaowengong	Alpine meadow	107°48′11.80″	33°59′24.32″	3350

**Table 2:** Correlation coefficients among diversity indices on the southern slope of Taibai Mountain.

Southern Slope	IND	SPE	H’	R	C	J
IND	1					
SPE	0.930 **	1				
H’	0.800 **	0.900 **	1			
R	0.890 **	0.990 **	0.940 **	1		
C	−0.330	−0.420 *	−0.430 **	−0.450 **	1	
J	0.319	0.330	0.570 **	0.360 *	0.170	1

NOTE: *N* = 35 for all analyses. IND, number of Individuals; SPE, Species richness; H’, Shannon–Wiener diversity index; R, Margalef’s richness index; C, Simpson’s dominance index; J, Pielou’s evenness index. Values are Pearson correlation coefficients. * Correlation is significant at the 0.05 level (2-tailed), *p* < 0.05; ** Correlation is significant at the 0.01 level (2-tailed), *p* < 0.01.

**Table 3:** Correlation coefficients among diversity indices on the northern slope of Taibai Mountain.

Northern Slope	IND	SPE	H’	R	C	J
IND	1					
SPE	0.890 **	1				
H’	0.780 **	0.910 **	1			
R	0.810 **	0.980 **	0.940 **	1		
C	−0.150	−0.160	−0.140	−0.180	1	
J	0.380 *	0.490 **	0.740 **	0.560 **	0.240	1

NOTE: *N* = 35 for all analyses. IND, number of individuals; SPE, species richness; H’, Shannon–Wiener diversity index; R, Margalef’s richness index; C, Simpson’s dominance index; J, Pielou’s evenness index. Values are Pearson correlation coefficients. *p* < 0.05; *p* < 0.01. Values are Pearson correlation coefficients. * Correlation is significant at the 0.05 level (2-tailed), *p* < 0.05; ** Correlation is significant at the 0.01 level (2-tailed), *p* < 0.01.

**Table 4:** Pairwise comparisons of Auchenorrhyncha species composition among different forest belts on the southern slope of Taibai Mountain. Values in the lower-left triangle indicate the number of shared species, whereas values in the upper-right triangle indicate similarity coefficients.

Southern Slope Zone	*Quercus* *variabilis*	*Quercus* *aliena*	*Betula utilis*	*Betula* *albosinensis*	*Abies fargesii*	*Larix potaninii*	Alpine Meadow
*Quercus variabilis*		0.39	0.18	0.12	0.04	0.05	0.02
*Quercus aliena*	29		0.26	0.19	0.06	0.06	0.02
*Betula utilis*	12	21		0.28	0.12	0.16	0.08
*Betula albosinensis*	8	13	12		0.25	0.15	0.10
*Abies fargesii*	2	4	5	5		0.27	0.44
*Larix potaninii*	2	4	6	3	3		0.36
Alpine meadow	1	1	3	2	4	3	

NOTE: Forest belts are arranged from low to high elevation.

**Table 5:** Pairwise comparisons of Auchenorrhyncha species composition among different forest belts on the northern slope of Taibai Mountain. Values in the lower-left triangle indicate the number of shared species, whereas values in the upper-right triangle indicate similarity coefficients.

Northern Slope Zone	*Quercus* *variabilis*	*Quercus* *aliena*	*Betula utilis*	*Betula* *albosinensis*	*Abies fargesii*	*Larix potaninii*	Alpine Meadow
*Quercus variabilis*		0.45	0.25	0.21	0.10	0.03	0.00
*Quercus aliena*	23		0.25	0.23	0.10	0.06	0.02
*Betula utilis*	11	16		0.15	0.12	0.03	0.00
*Betula albosinensis*	7	12	6		0.14	0.17	0.05
*Abies fargesii*	3	5	4	3		0.18	0.20
*Larix potaninii*	1	3	1	3	2		0.29
Alpine meadow	0	1	0	1	2	2	

NOTE: Forest belts are arranged from low to high elevation.

**Table 6:** Number of shared species (upper-right triangle) and similarity coefficients (lower-left triangle) among Auchenorrhyncha communities recorded in different mouths on the southern slope of Taibai Mountain.

Months	May	June	July	August	September
May		5	9	8	10
June	0.09		12	16	8
July	0.14	0.16		21	9
August	0.15	0.27	0.32		13
September	0.29	0.16	0.14	0.26	

**Table 7:** Number of shared species (upper-right triangle) and similarity coefficients (lower-left triangle l) among Auchenorrhyncha communities recorded in different mouths on the northern slope of Taibai Mountain.

Months	May	June	July	August	September
May		8	9	7	4
June	0.23		14	14	8
July	0.21	0.25		14	10
August	0.21	0.33	0.27		5
September	0.15	0.21	0.22	0.13	

## Data Availability

The original contributions presented in this study are included in the article. Further inquiries can be directed to the corresponding authors.
